# Exploring vagus nerve stimulation in postoperative delirium and dementia

**DOI:** 10.1186/s42234-026-00200-4

**Published:** 2026-02-20

**Authors:** Rachel M. Spicer, Peder S. Olofsson, Fiona E. Harrison

**Affiliations:** 1 Department of Medicine, Division of Diabetes, Endocrinology and Metabolism, Vanderbilt University Medical Center, Nashville, TN USA; 2 Department of Medicine, Laboratory of Immunobiology, Center for Bioelectronic Medicine, Division of Cardiovascular Medicine, Solna, Karolinska Institutet, Stockholm, Sweden

**Keywords:** Delirium, Delirium superimposed on Dementia, Vagus Nerve Stimulation, Alzheimer’s Disease, Neuroinflammation, Microglia

## Abstract

Microglial activation and neuroinflammation, important aspects of neurodegeneration and accumulation of amyloid-pathology, is often exacerbated by peripheral inflammation following surgical procedures. Subsequent postoperative delirium is a predictor for long-term cognitive decline and increased rick of Alzheimer’s Disease, and perioperative strategies to reduce inflammatory responses, may be a potential avenue to mitigate postoperative complications. In this issue of Bioelectronic Medicine, Song et al. utilize percutaneous vagus nerve stimulation (pVNS) as a potential novel avenue for the attenuation of neuroinflammation and postoperative cognitive decline, which we have discussed in this commentary.

## Main text

The term ‘delirium’ has been used for at least 2000 years. Coming from the Latin ‘Delirare’ which means ‘to be off the track’, delirium was coined by Aulus Cornelius Celsus (ca 25–50 BC) to describe general mental disorders, however, may have been also described by *Corpus Hippocraticum* when he described two mental disorders with high fever and physical illness: “phrenitis (agitation)” and “lethargus (lethargy)” (Deksnytė et al. 2012; Iglseder et al. 2022). The first definition of delirium that arguably refers to the delirium as it is considered today, came from Philip Barrough (1583), where he referred to it as a derangement of imagination, cognition and memory (Barrough (Barrough n.d.); Lipowski 1991). In 1959, Engel and Romano reframed it as a disturbance of consciousness with electroencephalographic slowing that correlated with the degree of cognitive impairment (Engel and Romano 1959). Today, the *Diagnostic and Statistical Manual of Mental Disorders, Fifth Edition (DSM‑5)* defines delirium as an acute disturbance in attention and awareness with a fluctuating course that may be accompanied by a change in cognition or perception, caused by a medical diagnostic criteria that cannot be better explained by another disorder, such as dementia (DSM-5™. 2013; Slooter et al. 2020).

Postoperative delirium is a predictor for long-term cognitive decline and increased risk of Alzheimer’s Disease and related dementias (ADRD). Surgical procedures often have pro-inflammatory effects that promote glial activation, an important aspect of neurodegeneration, Aβ accumulation, and delirium pathophysiology (Fruhwürth et al. 2024; Hansen et al. 2018). Delirium superimposed on dementia (DSD) refers to periods of delirium occurring concurrently with pre-existing dementia. DSD is more likely to occur in older populations, often also impacted by chronic neuroinflammation, and who are approximately eight times more likely to develop delirium during hospitalization and have an approximate two‑fold increase in two‑year mortality as compared with individuals without known cognitive decline (Francis and Kapoor 1992; Avelino-Silva et al. 2017; Fick, et al. 2007). Perioperative strategies that attenuate inflammatory responses may, therefore, be interesting avenues to explore for potential mitigation of postoperative delirium and cognitive decline.

There is currently no approved pharmacologic prophylaxis for delirium and current practice largely repurposes licensed agents to treat psychiatric symptoms or perioperative complications. For example, Parecoxib, a cyclooxygenase (COX)−2 selective non-steroidal anti-inflammatory (NSAID) has been reported to lower IL-1β and TNFα, and Dexmedetomidine, an α2-adrenoceptor agonist, may reduce the risk of postoperative cognitive decline (Alam et al. 2018). Unfortunately, the clinical evidence to date is heterogeneous, there is a lack of consensus on best-practice treatment, and there is accordingly a need for improved strategies for the vulnerable individuals at increased risk of post-operative cognitive impairment.

As published in *Bioelectronic Medicine,* Song and colleagues performed orthopedic surgery or intraperitoneal (I.P.) injection of IL-6 in the fast-progressing transgenic 5xFAD TG model and the more chronic-developing CVN-AD (APPSwDI/mNos2^−/−^) AD model, to investigate the effects of ultrasound-guided percutaneous vagus nerve stimulation (pVNS) on Aβ plaque-associated pathology (Song et al. 2026). Mice subjected to pVNS showed reduced hippocampal Aβ accumulation, TUNEL^+^ staining, and improved microglial morphology as compared with sham-treated mice.

During early-stage disease, Aβ oligomer aggregation drives microglial activation and expression of pro-inflammatory markers, which subsequently biases microglia to roles that include compaction, engulfment and elimination of plaques (Fan et al. 2017; Kinney et al. 2018; Miao, et al. 2023). However, continuous, or uncontrolled microglial activation during later stages of disease, or from secondary challenges, can result in aberrant responses to Aβ, exacerbated plaque accumulation and reduced capacity to clear Aβ (Fan et al. 2017; Kinney et al. 2018; Miao, et al. 2023). It is well documented that sepsis, endotoxemia, and/or orthopedic surgery, increases the number of Iba1^+^ microglia and promote morphological changes associated with an activated phenotype of microglia (Vo et al. 2025; Batista et al. 2019; Zhang et al. 2016). Earlier, simplistic conceptualization of microglia as beneficial or pro-inflammatory (termed M1 and M2) has developed to recognition of much more complex microglial profiles based on advanced methodologies and analysis, including single-cell transcriptomics (Paolicelli et al. 2022; Mills, et al. 2000; Nahrendorf and Swirski 2016; Jendresen et al. 2019). Data from the recent study by Song et al. capture some of this range of changes via morphology and expression of transcriptional markers that may also help in recognition of altered response of these cells across disease and pathology stage, as well as in the more acute response to surgery and vagus nerve stimulation. In fact, pVNS effectively rescued surgery-induced microglial changes in these mice, which may directly contribute to the cellular response to amyloid pathology observed post-surgery (Song et al. 2026).

In the context of post-operative cognitive impairment, on one hand, many studies agree that systemic inflammation can induce neuroinflammatory changes including microglial activation and aberrant gene expression changes, and inflammation has been associated with delirium and poor long-term cognitive decline (Wang et al. 2018; Giridharan et al. 2023; Basak et al. 2021; Denver and Cunningham 2025; Li 2022; Xiao et al. 2023). On the other hand, there is still a limited understanding of the mechanistic details of the interaction between markers of inflammation, microglial activation, Aβ plaque accumulation, and neuroinflammation for long-term cognitive impairment and dementia in the perioperative setting. Whether Aβ burden is increased following acute challenge may depend on experimental models and design and thus reflect a more nuanced pattern of changes rather than a clear pathway to increased pathology (Zhang et al. 2016; Jendresen et al. 2019; Li 2022; Xiao et al. 2023; Othman et al. 2023). The study by Song et al. opens a potential avenue for peripheral nerve activation in attenuation of neuroinflammation and postoperative cognitive decline. However, the time course and functional phenotypes of microglial activation underlying perioperative cognitive impairment are not yet well delineated. Song and colleagues showed that treatment using the minimally invasive pVNS attenuated a delirium-like phenotype in AD-vulnerable mice, supporting the notion that further exploration of the activity of the vagus nerve in the perioperative setting may be important and potentially identify suitable approaches for reducing the risk of surgery-associated cognitive decline in at-risk individuals. To advance pVNS, future studies should integrate time‑resolved imaging, single‑cell transcriptomics, and targeted neuromodulation to map microglial–neuronal interactions with higher resolution. We anticipate that this work will strengthen the mechanistic framework and support responsible clinical translation.

## Data Availability

Not applicable.

## Abbreviations

Aβ: Beta-amyloid
AD: Alzheimer’s Disease
ADRD: Alzheimer’s Disease and Related Dementias
DSD: Delirium superimposed on dementia
LPS: Lipopolysaccharide
pVNS: Percutaneous vagal nerve stimulation
